# The Efficacy and Safety of Biologics in Treating Ankylosing Spondylitis and Their Impact on Quality of Life and Comorbidities: A Literature Review

**DOI:** 10.7759/cureus.55459

**Published:** 2024-03-03

**Authors:** Abdulrahman Alotaibi, Danah Albarrak, Yousef Alammari

**Affiliations:** 1 College of Medicine, Imam Mohammad Ibn Saud Islamic University, Riyadh, SAU; 2 College of Medicine, King Saud Bin Abdulaziz University for Health Sciences, Riyadh, SAU

**Keywords:** quality of life, biologics, ankylosing spondylitis, safety, efficacy

## Abstract

Ankylosing spondylitis (AS) is a chronic inflammatory arthritis that affects the axial skeleton, causing intense pain, progressive joint destruction, and a gradual reduction in physical function. Additionally, AS can result in extra-musculoskeletal manifestations including inflammatory bowel disease (IBD), psoriasis, and acute anterior uveitis (AAU) affecting patients' quality of life (QoL). Furthermore, AS association with neurological and cardiovascular events has been documented. With the advent of biologics, treating AS has dramatically changed due to their high efficacy and tolerable safety. Nevertheless, there are differences in traits, including rapidity of onset, long-term efficacy, safety profile, and influence on comorbidities. A better understanding of such traits enables clinicians to make the best decision for each patient, increasing persistence, extending medication survival, enhancing patient satisfaction, and reducing the disease effect of AS. A review of the literature published in English in PubMed and Google Scholar databases from 2010 to 2023 was conducted. All relevant results fitting the scope of the topic were included. In this article, we emphasize biologics' efficacy and safety profile in patients with AS. In addition, we discuss the impact of biologics on comorbidities and health-related quality of life (HRQoL).

## Introduction and background

Background on ankylosing spondylitis (AS)

Ankylosing spondylitis (AS) is a classic inflammatory arthritis that affects the axial skeleton. It is characterized by acute and chronic inflammation causing intense pain, progressive joint destruction, and a gradual reduction in physical function [1-3]. AS is a multifactorial condition with a known hereditary cause HLA-B27 genotype (human leukocyte antigen) [2], and it is also known to be a prototype disease for a class of conditions known as spondyloarthritis (SpA). These illnesses share clinical, radiological, and genetic characteristics, including an increase in the rates of inflammatory bowel disease (IBD), psoriasis, and acute anterior uveitis (AAU), ultimately known as extra-musculoskeletal manifestations [3]. AS affects males more commonly and typically manifests in the second and third decade of life and rarely occurs after the age of 45 [4]. It is difficult to precisely state the prevalence due to the lack of literature compared to other rheumatic diseases; however, the prevalence of AS has been estimated to range between 0.1% and 1.4% globally [5]. Moreover, a systematic review by Dean et al. [6] attempted to illustrate the global prevalence of AS in Europe, Asia, North America, Latin America, and Africa. The reported frequency of AS varies significantly between continents, but there is some consistency within these regions. Compared to Latin America (mean: 10.2, weighted mean: 12.2 per 10,000), AS is more prevalent in Europe (mean: 23.8, weighted mean: 18.6 per 10,000) and Asia (mean: 16.7, weighted mean: 18.0 per 10,000). The systematic review, which aimed to estimate the global prevalence of AS in North America and Africa, reported 31.9 and 7.4 AS cases per 10,000 people, respectively. Additionally, the anticipated number of AS cases in Europe and Asia ranges from 1.30 to 1.56 million and 4.63 to 4.98 million, respectively. In all investigations, the average gender ratio is 3.4:1. (males:females) [6]. Chronic inflammation progressively causes the vertebral column to ossify, which results in substantial impairments in physical function and spinal movement, and ultimately impacts the quality of life (QoL) [7]. The main focus of this literature review is to discuss the efficacy and safety of biologics in treating AS and their impact on quality of life and comorbidities.

Pathogenesis

AS develops as a result of complex interplay that remains partially understood. However, certain factors have been identified to play an essential role in its pathogenesis, including genetics, certain infections, environmental exposure, and sex hormones. Based on emerging evidence, the pathophysiological response in AS has been identified as a combination of auto-inflammatory and autoimmune processes [8]. AS has a vital genetic component with high monozygotic twin concordance and a high heritability rate of 63% and 90%, respectively [9,10]. The presence of major histocompatibility complex (MHC) class I allele HLA-B27 accounts for significant genetic risk, which is found in more than 90% of patients with AS [11]. HLA-B27 has been hypothesized to result in a pro-inflammatory effect in multiple ways, including the presentation of arthritogenic peptides, NK receptor recognition of HLA-B27 cell surface dimer, and HLA-B27 misfold during biosynthesis [12,13]. However, a study conducted in 2014 compared AS and non-AS-associated HLA-B27 subtypes and suggests that disease-associated alleles have increased intracellular aggregates of MHC misfolded protein, the functional impact of which is still unclear, with lack of an explicit endoplasmic reticulum (ER) stress [14]. Undisputedly, HLA-B27 plays a critical role in AS pathogenesis. However, recent estimates propose that it only accounts for 20%-25% and 40% of the total heritability and genetic risk, respectively [10,15]. In non-HLA-B27, genome-wide association studies (GWASs) have identified common single nucleotide polymorphisms (SNPs) to have a highly significant association with AS [16-18]. Interestingly, many of these genes are found along distinct immunomodulatory pathways [15,19]. In particular, multiple genes affect the development and activity of Th17, a recently identified population of T helper cells named so for their production of interleukin (IL)-17 [20]. Th17 cells develop from naive T cells under conditions where the expression of IL-23 receptor (IL-23R) is induced by transforming growth factor-β (TGF-β) and pro-inflammatory cytokines such as IL-6 and IL-1β. However, for Th17 cells to become pathogenic, they require IL-23, which may be produced by innate immune cells including macrophages and dendritic cells [21]. GWAS has identified genes that influence the IL-17/IL-23 pathway, including cytokines and cytokine receptors (IL-23R, IL-12B, IL-6R, IL-1R1, IL-1R2, and IL-27), signaling molecules downstream of the IL-23R (JAK2, STAT3, and TYK2), and gene products transducing signals from infectious stimuli [18].

Therapeutic targets

The IL-17/IL-23 pathway remains the major pathway in AS. Consequently, the IL-17 axis is the established target of AS therapy, although the IL-23/Th17 axis has lately received interest as a potential inflammatory mechanism. Innate immune cells that generate IL-17 are more prevalent during inflammation. IL-17A and IL-17F, two members of the IL-17 cytokine family, show 50% structural similarity and comparable pro-inflammatory properties and communicate via the same receptor complex. IL-17A and IL-17F can be specifically neutralized by the monoclonal antibody known as bimekizumab [22,23]. In order to induce and maintain Th17 cells, IL-23 is a crucial factor [24,25]. Studies have demonstrated a link between AS risk and IL-23 receptor (IL-23R) polymorphism. Additionally, studies have indicated that IL-23 contributes to disease pathophysiology [26,27]. The clinical hypothesis that direct and specific suppression of IL-23 will have therapeutic effects in patients with AS is supported by using IL-17A inhibitors (such as secukinumab) in treating AS.

## Review

Methodology

To identify articles related to the efficacy and safety of biologics in treating ankylosing spondylitis and their impact on quality of life and comorbidities, we performed a database search using PubMed and Google Scholar databases. Results were limited to articles published between January 2010 and June 2023 and written in English. The initial search resulted in 720 articles, and we screened 192 of them for title and abstract. Finally, 60 publications were included in the literature review. Papers that did not fit the scope of the study and did not investigate the efficacy and safety of biologics, case reports and case series, and studies not published in English were excluded.

Data on the use of biologics to treat ankylosing spondylitis have been accumulated so far. The effectiveness and safety profile of biologics in the treatment of ankylosing spondylitis are the main topics of this research, as well as its effects on comorbidities and the quality of life of patients.

Efficacy and safety of biologics in treating AS

The European Alliance of Associations for Rheumatology (EULAR) recommends nonsteroidal anti-inflammatory drugs (NSAIDs), biological disease-modifying antirheumatic drugs (DMARDs), non-biological DMARDs, analgesics, glucocorticosteroids, non-pharmacological treatments (such as education, exercise, and physical therapy), and surgical intervention to treat AS complications. However, there is no proof that non-biological DMARDs are effective in axial illness [28]. Biological DMARDs have become the mainstay of therapy in certain cases of AS due to their clear benefits in working rapidly and efficiently compared to non-biological DMARDs. Compared to rheumatoid arthritis (RA), the selection of biologics for AS is more limited [28]. For the treatment of AS, only IL-17 and tumor necrosis factor (TNF) inhibitors (TNFis) have successfully met primary objectives in clinical trials [28,29], making them the current standard of care in biological therapy. Adalimumab (ADA), certolizumab (CZP), etanercept (ETN), golimumab (GOL), and infliximab (INF) are the five TNF-α that have been authorized for use in patients with active AS [28]. A recent study demonstrated that TNF inhibits disease activity and shields the spine's structural integrity [30]. In addition, various evidence illustrates that the IL-17 pathway has been identified as an effective therapeutic target in treating AS [31-34]. Indeed, people with AS have higher levels of IL-17 and IL-23-producing cells in their circulation and target organs, making IL-17 inhibitors a primary therapeutic target [33-37].

Seven biologic medications have been extensively compared regarding efficacy, tolerability, and safety in treating AS using network meta-analyses based on high-quality randomized controlled trials (RCTs). These biologics are IL-6 inhibitor (i.e., tocilizumab), IL-17A inhibitor (i.e., secukinumab and ixekizumab), IL-17A/F inhibitor (i.e., bimekizumab), IL-23 inhibitor (i.e., risankizumab and ustekinumab), JAK inhibitor (i.e., upadacitinib and tofacitinib), TNF-α inhibitor FC fusion protein (i.e., etanercept), and TNF-α fully human monoclonal antibody (i.e., infliximab, adalimumab, certolizumab pegol, and golimumab). Their principal findings could be summarized in three points. First, the IL-17A/F dual variable domain inhibitor, which has the best effectiveness and safety, has the best chance of being the best therapeutic option. Second, TNFi had the most significant impact on reducing disease activity and enhancing functional capacity. Third, it was discovered that JAK inhibitors considerably outperformed a placebo in the primary network. According to the cluster rank analysis, INF is the safest and most effective biologic medication for treating AS, while IL-17 (bimekizumab) inhibitor is the most potent and well-tolerated biologic medication [38].

A meta-analysis was conducted by Deodhar et al. [39] to examine the relative efficacy of 11 kinds of IL-17A inhibitor, JAK inhibitor, and TNFi medications (i.e., adalimumab, certolizumab pegol, etanercept, filgotinib, golimumab, infliximab, ixekizumab, risankizumab, secukinumab, tofacitinib, and ustekinumab). The outcomes assessed were ≥20% improvement in the Assessment of Spondyloarthritis International Society Criteria (ASAS20), Bath Ankylosing Spondylitis Functional Index (BASFI), and C-reactive protein (CRP) at weeks 12-16. They found that tofacitinib 5 mg was the top-ranked treatment for ASAS20 response, followed by intravenous (IV) golimumab 2 mg/kg. IV golimumab 2 mg/kg and infliximab 5 mg/kg were the top two ranked treatments for change from baseline in BASFI and changed from baseline in CRP. Baeten et al. [40] conducted a trial to assess the efficacy of secukinumab (anti-IL-17A monoclonal antibody) in treating patients with active AS. A total of 371 patients were randomly assigned to receive secukinumab or a placebo. They concluded that at week 16, ankylosing spondylitis signs and symptoms were significantly reduced by secukinumab at a subcutaneous (SC) dose of 150 mg, with either subcutaneous or intravenous loading dose (10 mg/kg of body weight). A 75 mg subcutaneous dose of secukinumab significantly improved the patient's condition only when a more significant intravenous loading dose was used. Additionally, 30%-40% of the trial participants have had anti-TNF failed trials in treating AS. As a result, secukinumab might be helpful to individuals whose prior anti-TNF therapy was unsuccessful and in patients who have never received TNF medications [40]. Similar outcomes were shown for the secondary endpoints, which included a decrease in the score of the Bath Ankylosing Spondylitis Disease Activity Index (BASDAI), Ankylosing Spondylitis Quality of Life (ASQoL), Short-Form (SF)-36 health survey, and levels of C-reactive protein (CRP) [40]. Many complications have been documented in the study of Baeten et al. [40] and are relevant to previous studies regarding safety concerns about using secukinumab [38,39]. The incidence of infections or infestations was higher with secukinumab than with placebo. For the duration of treatment, patients receiving secukinumab experienced 0.7, 0.9, and 0.7 occurrences per 100 patient-years, respectively, of grade 3 or 4 neutropenia, *Candida* infections, and Crohn's disease [40]. The significant role that IL-17 plays in host defense against fungal infections, particularly in mucosal areas, is likely the cause of the increased occurrence of *Candida* infection.

In addition, compared to individuals using secukinumab to treat psoriasis or psoriatic arthritis, people with AS in secukinumab therapy exhibit a higher propensity to develop inflammatory bowel disease. However, this could be explained by the fact that a sizable proportion of AS patients have histological results that resemble those of inflammatory bowel disease [39,40]. Moreover, compared to patients with psoriasis or psoriatic arthritis, people treated with secukinumab for AS tend to get uveitis more frequently, which is most likely because anterior uveitis is the extra-articular symptom of AS that occurs most frequently [41]. Chen et al. [42] performed a network meta-analysis including 14 trials (2,672 patients) to compare the effectiveness of all biologics in treating active AS. Except for secukinumab and tocilizumab, most biological treatment regimens were more successful than placebo in all outcomes evaluated. There were no significant differences between biological treatments for AS, except that infliximab 5 mg was preferable to tocilizumab, given the probability that infliximab 5 mg/kg would be considered the best biological treatment for AS. At the same time, secukinumab had the highest probability of being ranked the second best [42]. Those findings were consistent with the comparison of Migliore et al. [43] between subcutaneous biologic agents in treating AS [43].

A retrospective study on Italian AS patients was done with 283 individuals. As the first anti-TNF medications, patients had received treatment with ADA (18.7%), ETN (26.8%), and INF (54.4%). This group had a very high partial remission rate (57.6%), and there were no discernible differences in the likelihood of achieving partial remission among TNF blockers [44]. TNF inhibitor usage was linked to a greater risk of herpes zoster than those who did not use disease-modifying antirheumatic medications in a population-based research conducted in South Korea on AS patients [45]. Regarding the development of neoplasms, most studies did not reveal evidence of a higher risk of malignancies among AS patients who used TNF inhibitors [46]. Another possible therapeutic target for AS is the JAK pathway, a new therapeutic class for immune-mediated inflammatory diseases. A selective JAK1 inhibitor called upadacitinib is being investigated for additional immune-mediated inflammatory conditions, including psoriatic arthritis, ulcerative colitis, Crohn's disease, and atopic dermatitis [47-49]. Axial spondyloarthritis may be treated by targeting JAK pathways, and two phase 2 studies have demonstrated the efficacy of JAK inhibitors (tofacitinib and filgotinib) in treating AS [50,51]. In a study done by van der Heijde et al. [51] to assess the efficacy of upadacitinib after 14 weeks of therapy, individuals with active AS experienced a marked improvement in the disease activity, function, and axial inflammation on magnetic resonance imaging (MRI) when given oral upadacitinib 15 mg once daily. Moreover, no fatalities, serious infections, herpes zoster, cancer, venous thromboembolic events, or malignancies were observed [52].

A study by van der Heijde et al. [53] investigated the efficacy of ixekizumab (a high-affinity monoclonal antibody that selectively targets IL-17A) and ADA. Subcutaneous (SC) ixekizumab 80 mg was given every two weeks for 83 patients and the same dose over four weeks for 81 patients, and the ADA group was given 40 mg SC over two weeks. The main goal was to compare the percentage of patients who achieved an ASAS40 response, a composite indicator of clinical improvement in axial spondyloarthritis. The result showed that more than half of the patients who received 80 mg SC for two weeks achieved ASAS40 compared to 48% of those who took the exact dosage for four weeks, while 36% on ADA achieved ASAS40 [53]. Similar to secukinumab, ixekizumab is an IL-17A antagonist. Ixekizumab and secukinumab, however, vary in several molecular and pharmacokinetic properties. For instance, ixekizumab's in vitro binding affinity to IL-17A is 18 pmol/L instead of secukinumab's 100-200 pmol/L, which can lead to extra safety or effectiveness profiles [54].

Impact of biologics on health-related quality of life (HRQoL) and comorbidities

Health-related quality of life (HRQoL) in AS patients has been reported to be decreased, especially in those with higher disease activity, functional disability, and peripheral involvement [55-57]. Consequently, with the introduction of biologics in treating AS, multiple studies aimed to assess their impact on patients and HRQoL. TNF-α blockers were found to have a possible antidepressant effect [58]. A 6-week longitudinal study conducted back in 2010 investigated the impact of TNF-α antagonist therapy on depression, anxiety, and quality of life (QoL) in AS patients. The authors concluded that mental health scores improved after the beginning of the treatment. In particular, a significant reduction in depression and anxiety scores was observed after the second and third infusions of infliximab, an anti-TNF treatment. Additionally, QoL scores reflected similar improvement as well [59]. Another study investigated the impact of TNF inhibitors on the risk of development of dementia in AS patients. Even though AS patients had a higher prevalence of Alzheimer's disease compared to the general population, among the patients with AS, those treated with TNF inhibitors had lower rates of Alzheimer's disease [60].

Multiple studies revealed the positive impact of biologics on HRQoL in AS patients. A recent study revealed that biological agents are superior in improving QoL in AS patients compared to those receiving only a placebo or standard therapy [61]. A 24-week randomized controlled study evaluated the impact of adalimumab on HRQoL in patients with active AS. Adalimumab significantly improved HRQoL and physical health in active AS patients throughout the 24 weeks of the study [62]. Furthermore, a three-year outcome-based randomized controlled trial concluded that adalimumab significantly improved physical function, disease activity, and HRQoL in AS patients [63]. Another study aimed to evaluate the effect of TNF inhibitors (namely, infliximab and etanercept) in AS patients over 65 years, which revealed significant improvement in HRQoL and functional disability [64]. Additionally, TNF inhibitors have been shown to reduce pain and fatigue in patients with AS, improving QoL [65]. Furthermore, another randomized controlled trial assessing the impact of certolizumab pegol on AS patients revealed improvements in pain level, fatigue, sleep, and HRQoL in both AS and non-radiographic axial SpA patients [66]. Moreover, a randomized, double-blind, placebo-controlled multicenter study conducted in 2020 aimed to investigate the effect of golimumab on HRQoL in AS for 28 weeks. The patients were randomized to receive golimumab or placebo at weeks 0, 4, and 12 and every eight weeks, with placebo crossover to golimumab at weeks 16 and 20 and every eight weeks. The results revealed that more remarkable improvement in HRQoL was observed early in the golimumab group at week 8 and throughout week 16 compared to the placebo group. After the placebo group crossover to golimumab at week 16, both groups reported similar HRQoL improvement at week 28 [67].

Furthermore, a more recent systematic review and meta-analysis aimed to investigate the impact of biological therapy, namely, TNF inhibitors or interleukin-17A (IL-17A) antibody agents, on HRQoL in AS patients, specifically those with radiographic axial spondyloarthritis (r-axSpA). The analysis included 16 randomized clinical trials; the results revealed an association between using biological agents and significant improvement in HRQoL among patients with r-axSpA [68]. A 12-week, double-blind, multicenter study conducted in 2007 assessed the effect of etanercept 50 mg once weekly versus 25 mg twice weekly on the QoL of AS patients. Both dosing regimens significantly improved patient function and QoL [69] in a randomized, open-label study aimed to assess HRQoL and the impact of etanercept therapy among AS patients. It was noted that patients with active AS reported a significant reduction in AS, especially across the physical domains. After the initiation of etanercept therapy, significant improvement in HRQoL was observed among AS patients, with tremendous improvements reported in physical domains [70]. Moreover, a 12-week double-blind, randomized control trial observed improvement in QoL of patients with active AS who received bimekizumab, an IL-17A/F inhibitor [71]. Additionally, compared to placebo AS, patients who received ixekizumab (IL-17A inhibitor) reported improvement in QoL, including pain level, fatigue, and sleep [72]. Furthermore, multiple studies investigated the impact of JAK inhibitors, namely, upadacitinib, and tofacitinib, on the QoL of AS patients. All concluded that treatment with JAK inhibitors significantly improved disease activity, QoL, and work productivity [73-75].

As stated earlier in the review, biologic DMARDs showed tolerable safety profiles. However, mounting data pointed to issues exclusive to certain drugs. TNF-α inhibitors have been linked to paradoxical reactions, lupus, severe infections (with a slightly elevated risk), tuberculosis, and psychiatric conditions such as anxiety and depression [55-57]. Inflammatory bowel disease, neutropenia, and candidiasis are linked to IL-17 inhibitors [33-37]. As of the present time, no adverse effects specific to IL-23 inhibitors have been documented [38]. The use of TNF-α inhibitors raised concerns about two major safety risks related to biologics: interstitial pneumonia and hepatitis B virus reactivation [38,39]. There is little evidence for more recent biologic DMARDs, such as IL-17 and IL-23 inhibitors, and observation with long-term comparable studies is needed to illustrate the safety and efficacy of biologics in treating AS.

## Conclusions

Our study offers a thorough narrative evaluation of the use of biological medicines in treating individuals with AS who may currently be treated with a wide range of biological medications. However, there are differences in traits, including rapidity of onset, long-term efficacy, safety profile, and implications on comorbidities. A better knowledge of such traits enables clinicians to make the best decision for each patient, increasing persistence, extending medication survival, enhancing patient satisfaction, and reducing the impact of AS on the quality of life.
